# How does feedback shared with interprofessional health care teams shape nursing performance improvement systems? A rapid realist review protocol

**DOI:** 10.1186/s13643-019-1097-2

**Published:** 2019-07-22

**Authors:** Joachim Rapin, Joanie Pellet, Cedric Mabire, Sylvie Gendron, Carl-Ardy Dubois

**Affiliations:** 1 Faculté des sciences infirmières, Université de Montréal, 2375 Chemin de la Côte-Sainte-Catherine, Montréal, Québec H3T 1A8 Canada; 2 Institute of Higher Education and Research in Healthcare – IUFRS, Faculty of Biology and Medicine, Lausanne University Hospital, University of Lausanne, Route de la Corniche 10, 1010 Lausanne, Switzerland; 3 École de santé publique de l’Université de Montréal, 7101 Avenue du Parc, Montréal, Québec H3N 1X9 Canada; 4 Vaud University Hospital, Rue du Bugnon 21, 1011 Lausanne, Switzerland

**Keywords:** Feedback, Nurses, Quality improvement, Quality of health care, Efficiency, Organisational, Outcome and process assessment, Health care, Review

## Abstract

**Background:**

Nursing care quality varies between hospitals, and even between departments within the same institution. Suboptimal care can have deleterious consequences for patients such as lengthened hospital stay, nosocomial infection, pressure ulcers or death. Experts recommend the implementation of nursing performance improvement systems to assess team performance and monitor patient outcomes and efficiency savings. In practice, these systems are expected to include feedback processes directed towards nursing teams and interprofessional staff in order to facilitate adjustments and improve their performance. Unfortunately, feedback appears somewhat haphazard and, at times, overlooked. This could be explained by an ongoing absence of clear recommendations. As a result, feedback effects are inconclusive: some teams improve their practice, others do not. Although feedback has been conceptualised and studied from different theoretical perspectives, ongoing empirical inconsistencies remain unexplained. The goal of this rapid realist review protocol is to develop a theory that explains how feedback shared with interprofessional health care teams shape nursing performance improvement systems.

**Method:**

This study follows standard guidelines established for realist reviews. Mechanisms at work will be analysed using Actor-Network Theory. All scientific documents are selected from five databases, are published in both English and French between 2010 and 2018, and include empirical research, reviews and grey literature. First, selection of documents will proceed on the basis of titles and abstracts; followed by a second selection by reading the remaining full texts. Inclusion criteria and a data extraction form will be pilot tested with 40 articles prior to completion by two reviewers. Data will be summarised in the form of [context, mechanism, outcome] equations to theorise operational feedback.

**Discussion:**

The innovative combination of Actor-Network Theory with a realist methodology holds promise for the identification of explanatory equations in complex systems and theory development. A rapid realist review is relevant to address an enduring knowledge gap which requires theory development. This preliminary study lays the groundwork for a pioneering theory on feedback in nursing performance improvement systems that will subsequently inform a multiple case study.

**Systematic review registration:**

Prospero CRD42018110128

**Electronic supplementary material:**

The online version of this article (10.1186/s13643-019-1097-2) contains supplementary material, which is available to authorized users.

## Background

Nursing quality, resource allocation, patient outcomes and care-related adverse events vary widely across contexts [1–5] and result in significant costs to patients and health systems [6–8]. As an example, over a 1-year period, Tchouaket et al. [8] identified 183 inpatients in 22 medical-surgical units in Québec who experienced at least one care-related adverse event which accounted for 1300 additional days of hospital stay at an extra cost estimated to CA$600,000, up to CA$2 million. To reduce these preventable human and social costs, experts recommend the implementation of performance improvement systems [9–11]. Nursing performance improvement systems (NPIS) have been implemented and evaluated for nearly 30 years [12, 13]. However, some processes in these systems have varying effects, or no effect, depending on the context.

Nursing performance is defined as “the capacity demonstrated by an organisation or an organisational unit to acquire the needed nursing resources and use them in a sustainable manner to produce nursing services that effectively improve patients’ conditions” ([14], p., 6). An NPIS is designed to measure a set of valid and reliable indicators relevant to nursing— e.g. changes in human resources, quality of care and patient outcomes—in order to assess the performance of nursing services and the effects of improvement initiatives [15, 16]. Doran et al. ([13], p., 10) define nursing-sensitive indicators as “relevant, based on nurses’ scope and domain of practice, and for which there is empirical evidence linking nursing inputs and interventions to the outcome”.

Several key processes are involved in an NPIS: choice of indicators, their operationalisation and validation, feedback to teams, analysis of results, and adjustments to improve practice [17]. Friedman et al. [18] conceptualise performance improvement systems as cyber-social systems, or Learning Health Systems, where both individuals and technologies are capable of self-learning and improvement. Cyber-social systems encompass five attributes: (1) they include data pertaining to characteristics and skills of a large number of individuals (e.g. professionals and patients) as well as other data (e.g. structural); (2) indicators help identify optimal care in support of judgement and actions of individuals; (3) self-learning and improvement are ongoing processes; (4) several simultaneous improvement processes can be identified and operated; and (5) stakeholders build and enact system values, which become part of their culture, through continuous learning and improvement activities [18].

According to Contandriopoulos et al. [17], NPIS are complex systems, otherwise described as open systems of organized action that are environment dependent. Complex system processes are driven by a significant number of interdependent actors who retain a certain degree of autonomy within the structure of the system that is, otherwise, open on its environment. In this respect, complex systems determine and are determined by their actors (and actions) which, in turn, depend on and (re)generate the system structure (organization, resources and values) that, recursively, may transform (or not) ongoing action and produce emergent outcomes [17]. Within such a system, both actors and structural entities thus have the potential to foster innovation [17], whilst emergent outcomes are explained by interactions between actors or contextual dimensions. Causality, therefore, is complex, in the form of multiple, non-linear, emerging, recursive causal chains; and similar results may follow different causal chains [17]. Ultimately, complex systems generate paradoxes; they evolve through antagonistic dynamics which create tension and which must be managed [e.g. creativity vs rules, autonomy vs dependence, desire to improve one’s practice vs difficulty in implementing it] [17]. In this research, and in line with Friedman et al. [18] and Contandriopoulos et al. [17], we have chosen to conceptualise NPIS as complex cyber-social systems.

Evaluation strategies that focus primarily on characteristics and outcomes of an action are subject to important limitations in the assessment of complex cyber-social systems; they do not help understand how such systems work and why their outcomes vary [19, 20]. It has been postulated that variable or unexpected outcomes can be explained by underlying (non-observable) mechanisms and structures, depending on the contexts in which they operate; and that exploration of these entities and their interactions can help understand how these complex systems work and, by extension, can improve their evaluation as well as the pertinence of results [19, 21, 22]. Specific entities and their interactions can be represented by causal chains to ultimately develop a system theory, sometimes referred to as a programme theory. This evaluative approach to complex systems can further be complemented by three principles that Bilodeau and Potvin [23] have derived from Actor-Network Theory (ANT) to theorise such systems as networks of interrelated entities: (1) investigate the connectivity processes between entities; (2) assume that individuals and other entities have their own abilities; and (3) presume that a network can (re)configure itself and evolve over time. Conceptualising an intervention as a complex system along ANT principles therefore suggests that trends in a network can be modelled chronologically to understand how the intervention evolves [23, 24]. Hence, we postulate that a realist approach to the evaluation of complex systems and ANT are not conflicting and that they could be combined to improve the understanding and the assessment of NPIS.

Despite current knowledge regarding performance improvement systems, it appears that provision of feedback on measured outcome indicators to interprofessional teams is an enduring challenge; the causal chains that could explain how feedback improves performance have not been clearly identified [25–27]. Therefore, once performance indicators are measured and calibrated (i.e. audit process), it remains unclear how to best share results with interprofessional teams (feedback process). In turn, teams’ capacities to analyse their results, develop action plans and modify their practice, if necessary [28], is impeded.

A Cochrane systematic review with multivariable meta-regression suggests that feedback slightly improves professional compliance with required clinical activities [28]. This compliance, however, is shown to vary greatly depending on the context and may also depend on initial performance of service systems and feedback provision modes [28]. Suggested improvements that could optimise team feedback effectiveness have been formulated [25, 26, 29]. However, these do not explain how feedback process could take place and evolve, nor do they provide any detail on interactions between involved entities or contextual influences. Notwithstanding the continued use of evaluation strategies that have major limitations, one explanation for the dearth of significant evidence resides in the scarcity of operational theories (i.e. middle range theory). Colquhoun et al. [30] note that fewer than 10% of studies conducted on audit and feedback interventions explicitly mention the use of a theory. When theories are mentioned: (a) they are poorly operationalised or appear to have been intuitively constructed [26]; and (b) they provide little explanation for the variability of outcomes [25, 26]. This omission makes it difficult to understand how feedback system entities interact with one another and with their context, and possibly limits any capacity to adapt and transpose feedback interventions into other contexts, never mind evaluate or interpret their outcomes [25–27, 31].

Some authors have summarised available theories that could potentially explain how feedback works, for instance, cognitive, educational, organisational, behavioural or knowledge dissemination theories [26, 30, 32]. Colquhoun et al. [26] have identified up to 28 theories. One recommendation could be to use these theories in research. Nevertheless, given their number, heterogeneity, insufficient operationalisation, and a growing stock of failed attempts to explain feedback intervention outcomes, some authors recommend prioritising a deeper understanding of underlying causal mechanisms and their interactions within the specificities of their context [25–27].

In their realist review of patient-reported outcome measures, Greenhalgh et al. ([27], p., 22) developed a logic model of “provider responses to performance data following feedback of ‘poor’ performance”. Their model includes features such as “perceived pressure to respond, trust data or not, identify areas of poor care, investigate cause and identify possible solutions” ([27], p., 22). The authors refer to a sequence of 10 different middle-range theories to explain the mechanisms and outcomes of feedback interventions with regard to patient-reported measures, such as media pressure theory, intrinsic motivation theories, and peer review theories by Hibbard et al. [33]. Are these results applicable to an NPIS? Greenhalgh et al. assert that many mechanisms exist and could interact to explain what motivates individuals and organisations to improve patient care [27].

Several recent studies suggest that nursing-sensitive indicators play a critical role in the overall performance of health services and systems [9, 12, 13]. However, more evidence is required, overall, to enhance technical system devices for data collection and to further optimize social system processes in order to facilitate greater access to, as well as the use of, system performance indicators by health care teams [9, 16]. More refined conceptualizations of causal chains are needed to better understand feedback interventions in NPIS and explain outcomes. This, in turn, should improve ongoing evaluation and feedback system development initiatives [25–27, 31]. A rapid realist review can set the ground to meet this challenge. In particular, this method can support the development of an operational feedback theory to interprofessional teams which circumscribes a network of relevant interactions between contexts and mechanisms to explain outcomes [34–36]. To the best of our knowledge, no realist review or evaluation has been conducted either on NPIS or their feedback system.

This rapid realist review protocol is described below to conceptualise how feedback shared with interprofessional health care teams shape NPIS.

## Method

Realist reviews are rooted in realist philosophy [37]. Realism combines three main assumptions: (a) observable phenomena can be explained by one or more underlying mechanisms and the contexts in which they operate; (b) these phenomena are socially constructed, so that how they are understood varies from one individual to another; and (c) researchers seek to find the best possible explanation for observed phenomena by abduction [38, 39]. The intention is not to develop universal laws but to develop operational theories that offer compelling rationale [37].

The realist review method proposed by Pawson et al. [31] derives from the work of Pawson and Tilley [40] who came up with the equation [Context + Mechanism = Outcome (CMO)] to model causal chains and their outcomes. In this review, we will use the equation suggested by Byrne [34] [Context and Mechanism(s) = > Outcome]. Contrary to Pawson and Tilley’s equation, which indicates that each context adds up with only one mechanism to produce a result, Byrne’s equation suggests that context interacts with various mechanisms in a directional causal pathway to generate outcomes. Research on complex and interlinked interventions suggest long intertwined causal chains that include plural mechanisms and produce outcomes that reflect transient states [34]. Byrne’s equation, therefore, is congruent with our conceptualization of complex systems [17] and our approach to NPIS as cyber-social systems [18].

### Key concepts

The intervention under study is the feedback system of NPIS, otherwise referred to as audit and feedback intervention, which provides results pertaining to indicators that are sensitive to nursing care in specific populations. This review may also include feedback on patient safety indicators likely to be influenced by nursing care as well as any other activity related to NPIS feedback. There exists a large range of nursing-sensitive indicators related to resources, processes and patient results [9, 14]; or indicators influenced by professional compliance with desired practices [i.e. processes] [28]. In this review, we will focus on the 51 nursing care indicators suggested by Dubois, D'Amour [14].

The population of interest consists of nurses receiving NPIS feedback as well as any other individuals involved in this intervention. For the purpose of this review, the intervention context has been limited to hospitals, including outpatient services and residential facilities. This choice is justified by the current state of evidence in the field of nursing performance systems, since NPIS have mainly been developed in these settings compared to other locations [12]. For our purposes, hospitals

“… are health care institutions that have an organised medical and other professional staff, and inpatient facilities, and deliver services 24 hours per day, 7 days per week. They offer a varying of acute, convalescent and terminal care using diagnostic and curative services.” ([41], p., 1)

Outpatient services are generally part of ambulatory services provided in university and regional hospitals as well as in clinics, and may also include emergency and telehealth services [42]. Residential facilities are defined here as “long-term care facilities which provide supervision and assistance in activities of daily living with medical and nursing services when required.” ([43], p., 1)

### Aims

NPIS include a feedback system to share performance indicator results with nurses and other members of interprofessional health care teams. This rapid review aims to conceptualise an operational theory to explain what and how feedback commits these teams to improve their performance.

### Design

This rapid realist review will be driven by six steps, as recommended by Pawson et al. [31] and Wong et al. [44]: (1) initial theory development, (2) search strategy, (3) selection and appraisal of documents, (4) data extraction, (5) analysis and synthesis, and (6) presentation and dissemination of revised theory. Wong et al. [44] have suggested that these steps can also be applied to conduct rapid realist reviews; whereas they appear compatible with those proposed by Saul et al. [45]. Conducted over a 6-month period, as a preliminary phase to a realist evaluation that will be conducted in a Swiss teaching hospital, this proposal meets the criteria for a rapid realist review [35]. The PRISMA-P 2015 checklist by Moher et al. [46] is provided in (Additional file 1).

#### Step 1: Initial theory development

A first literature review was conducted by JR in order to substantiate the background information and to identify potential middle range theories presented in an earlier section of this paper. This phase relied on different databases (e.g. CINAHL, PubMed, Google Scholar) and searching through relevant articles (snowball strategy). Given the heterogeneity of available theories [26, 30, 32], their limitations in explaining outcomes [25, 26], and the scarcity of operationalized conceptualizations [26], we opted for an inductive approach to identify [Context and Mechanism(s) = > Results] equations and develop an original and operational feedback theory. To that end, we will apply ANT concepts that were initially developed by Callon [47] and Latour [48] to guide our literature review and ensuing theory development. ANT may be used to [1] conceptualise how a complex intervention can develop and evolve within a socio-technical network and (2) provide reflective tools to delineate and represent causal chains that produce observed changes or results [23]. In that respect, ANT should assist with the identification of mechanisms, contexts and their interactions. The final section of this paper will discuss the combination of ANT with a realist methodology. These theoretical conceptions are used to clarify the contexts, mechanisms and outcomes, as well as their interactions.

#### Step 2: Search strategy

Booth et al. [49] and Wong et al. [44] suggest that the literature search be conducted in two-joint phases. The first should aim to identify a logical model and middle range theories to explain the causal chains at work. The second phase is directed at the selection of articles (studies, review articles, concept papers, research reports and other relevant grey literature, websites or project initiation documents, for example) to test potential middle range theories [44]. The logic model developed by Greenhalgh et al. [27] will serve as a basis for this review.

At this stage, we will seek scientific documents about nursing-sensitive performance indicators, feedback processes and organisational change. The search will be carried out in the following databases: CINAHL, EMBASE, MEDLINE, Google Scholar (for grey literature) and Web of Science (for a snowball strategy). Table 1 presents the search strategy used in CINAHL. Studies published both in English and French, between January 2010 and the search date will be included.

**Table 1:** Search strategy in CINAHL

PICO	CINAHL
Population of interest (P)	(MH “Nurses+”) OR (MH “Nursing Care+”) OR (MH “Nursing as a Profession+”) OR (MH “Nursing Role”) OR TX nurs* AND
Issue of interest (I)	(MH “Organizational Development”) OR (MH “Feedback”) OR TI ( Feedback* OR “feed-back*” ) OR AB ( Feedback* OR “feed-back*” ) OR MW ( Feedback* OR “feed-back*” ) AND
Comparison of interest (C)	N/A
Outcome of interest (O)	(MH “Organizational Efficiency”) OR (MH “Quality of Health Care”) OR (MH “Clinical Governance”) OR (MH “Quality Management, Organizational”) OR (MH “Quality of Nursing Care”) OR (MH “Accountability”) OR (MH “Clinical Effectiveness”) OR (MH “Guideline Adherence”) OR (MH “Outcomes (Health Care)”) OR (MH “Nursing Outcomes”) OR (MH “Outcome Assessment”) OR (MH “Treatment Outcomes”) OR (MH “Quality Assurance”) OR (MH “Quality Assessment”) OR (MH “Quality Improvement”) OR (MH “Clinical Indicators”) OR (MH “Outcome Assessment Information Set”) OR (MH “Joint Commission Core Measures”) OR (MH “Health Plan Employer Data and Information Set”) OR (MH “Nursing Audit”) OR (MH “Peer Review”) OR (MH “Process Assessment (Health Care)”) OR (MH “Root Cause Analysis”) OR (MH “Variance Analysis”) OR (MH “Program Evaluation”) OR (MH “Benchmarking”) OR (MH “Accreditation+”) OR (MH “Organizational Change”) OR (MH “Diffusion of Innovation”) OR (MH “Performance Measurement Systems”) OR TI ( ((Perform* OR quality) N2 (measur* OR manag* OR improv* OR indicator* OR program* OR system* OR assess* OR evaluat* OR care OR health* OR organi?at*)) OR (Outcome? N2 (assess* OR evaluat* OR nurs* OR treatment OR care OR health*)) OR (clinical* N2 (indicator* OR governance OR effective* OR efficien*)) OR (organi?at* N3 (effective* OR efficien* OR change OR innovation)) OR (guideline* N2 adher*) OR “joint commission” OR “JCAHO” OR “Health Plan Employer Data and Information Set” OR “HEDIS” OR audit* OR “peer review*” OR (“process assessment” N2 health*) OR “Root Cause Analysis” OR “Variance Analysis” OR (program* N2 evaluat*) OR (organi?ation* N2 accountabilit*) OR benchmarking ) OR AB ( ((Perform* OR quality) N2 (measur* OR manag* OR improv* OR indicator* OR program* OR system* OR assess* OR evaluat* OR care OR health* OR organi?at*)) OR (Outcome? N2 (assess* OR evaluat* OR nurs* OR treatment OR care OR health*)) OR (clinical* N2 (indicator* OR governance OR effective* OR efficien*)) OR (organi?at* N3 (effective* OR efficien* OR change OR innovation)) OR (guideline* N2 adher*) OR “joint commission” OR “JCAHO” OR “Health Plan Employer Data and Information Set” OR “HEDIS” OR audit* OR “peer review*” OR (“process assessment” N2 health*) OR “Root Cause Analysis” OR “Variance Analysis” OR (program* N2 evaluat*) OR (organi?ation* N2 accountabilit*) OR benchmarking ) OR MW ( ((Perform* OR quality) N2 (measur* OR manag* OR improv* OR indicator* OR program* OR system* OR assess* OR evaluat* OR care OR health* OR organi?at*)) OR (Outcome? N2 (assess* OR evaluat* OR nurs* OR treatment OR care OR health*)) OR (clinical* N2 (indicator* OR governance OR effective* OR efficien*)) OR (organi?at* N3 (effective* OR efficien* OR change OR innovation)) OR (guideline* N2 adher*) OR “joint commission” OR “JCAHO” OR “Health Plan Employer Data and Information Set” OR “HEDIS” OR audit* OR “peer review*” OR (“process assessment” N2 health*) OR “Root Cause Analysis” OR “Variance Analysis” OR (program* N2 evaluat*) OR (organi?ation* N2 accountabilit*) OR benchmarking ) AND
Timeframe	2010–current
Other limiters	Language: English, French

As expected in realist methodology, more specific searches may be carried out during the review to test potential middle range theories which appear pertinent for the developing feedback theory [44, 49]. Should this occur, these particular searches will be presented in the final report in a table of research findings as well as in a research agenda [49].

### Inclusion criteria

No restrictions will be imposed on the study designs of the research articles included [44, 49]. The intervention, the population and the context, as defined in the “Key concepts” section, will be used as inclusion criteria.

Moreover, one of the following criteria must also be met to include a document in this review: (a) feedback to interprofessional teams is examined through the lens of a middle range theory or theoretical concepts; and (b) the paper provides empirical data to refine or test the NPIS feedback theory under development, especially in terms of context, mechanisms or outcomes [49]. Documents pertaining to individual feedback only will be excluded.

#### Step 3: Selection and appraisal of documents

Selection of documents, literature search and data extraction will be performed concurrently and iteratively [44]. The selection process will proceed as follows: (a) preliminary selection will be based on the title and summary of each document (by JR); and (b) final selection will be based on a comprehensive reading of the articles (by JR and JP), both in accordance with the above inclusion criteria. Reasons for exclusion will be documented in this second step.

A form describing the selection and appraisal process will be developed for this review; and will be tested and improved, if necessary, by two reviewers (JR and JP). Both reviewers will read 40 articles, complete a selection/appraisal form separately, then compare their results. This template will include the following information: article number and full reference, source database, the country where the study was carried out, reasons for inclusion or exclusion and quality assessment as per the criteria indicated below.

### Quality assessment

Quality of the selected documents will be assessed by two reviewers (JR and JP) according to two criteria: (a) relevance to the subject matter; and (b) scientific rigour [44]. The latter will comply with the reliability criteria proposed by Wong [50]:

“Trustworthiness of data assumes that the data have been obtained empirically with some sort of method(s) and so are unlikely to be simply fabricated; where it is unclear if any methods have been used to obtain data, treat them with scepticism; and always try to find more than one source of data that is relevant to an aspect of programme theory.” ([50], p., 178).

When a document will be considered for exclusion because of insufficient quality, the same reviewers (JR and JP) will discuss the choice. Should there be any disagreement, the entire team will engage in a discussion. When applicable, specific check-lists will be used [e.g. CASP] [27]. When it is agreed that the quality of a document is deemed to be insufficient, it will be excluded.

Lastly, all included articles will be examined by a pair of two reviewers from the team (JR, JP, CM, SG or CAD) in order to ensure greater reliability in their appraisal for inclusion. Should there be any disagreement, the entire team will engage in a discussion.

#### Step 4: Data extraction

Two reviewers (JR and JP) will extract the review data into a Microsoft Excel 2016® database developed for this purpose. Extraction will mainly be achieved through a selection of text excerpts [31]. The data extraction form will be tested (JR and JP) on 10 articles. Extracted excerpts will be compared to help refine the template which, in addition to previously documented information (Step 3), will include categories that correspond to ANT entities: who are the identified actors? What are their roles, interests and interactions? What do they know about feedback? What are the material or symbolic devices (e.g. values, norms) and their attributes? Is there an indication of strategic action? Have power dynamics been identified? Is there evidence of feedback system restructuration or adaptation? Have any focal or controversial issues been reported? Furthermore, we will identify the translation processes as defined by ANT: problematization, interessement, enrolment and mobilization.

We also plan to indicate which references, including websites, could be useful to test our developing middle-range theories. This being said, we do not anticipate that all sections in the extraction form will be completed, since the contribution of each document to the final operational theory can vary. All working documents, including selection/appraisal and extraction forms, will be uploaded on Google Drive® to ensure data sharing, study tracking and safety backups. Reviewers (JR, JP, CM, SG or CAD) will work in pairs to improve the reliability of the extracted data and describe the contribution of each document included in the final theory. Should disagreements arise, the whole team will engage in a discussion to fine-tune the data extraction process. If needed, we may exceptionally consult the authors. The final report will include a description of the contribution of each document from which data was extracted for this review.

#### Step 5: Analyses and synthesis

Data analyses will focus on interactions between contexts and mechanisms that could explain given outcomes. Once potential causal chains likely to explain NPIS feedback outcomes have been identified, they will be synthesised with reference to the logic model developed by Greenhalgh et al. [27]. This logic model will provide a template to connect actions with structural entities and outcomes. Each causal chain component will be empirically tested and refined to generate explanatory leads that may or may not be supported by known middle range theories [51]. In the latter case, realist methodology claims the essential role of scholarly imagination in the development of potential middle range theories [51]. To test our explanatory leads (e.g. our equations or potential feedback theories), Wong [50] recommends two criteria that are inherent to abductive thinking in realist epistemology: plausibility and consistency. Plausibility is defined here as “the best explanatory theory” given the state of our knowledge [50]. The following criteria will be used to assess the consistency of our potential feedback theories: “consilience (or explanatory breadth)—the ability of the theory to explain as much as possible of the data; simplicity—the theory is simple and does not have to have special (or ‘ad hoc’) assumptions made to it to explain data; analogy—the theory fits in with what we currently know and/or substantive theory.” ([50], p., 179).

Additionally, since the same mechanisms and contexts can generate different outcomes [31], we expect that it will be necessary to explain such apparent discrepancies between equations; or that we will re-assess different middle-range theories. For instance, feedback from the public and confidential input can achieve different outcomes in a given context [27]. In the end, plausible and consistent theory incorporating the equations [Context and Mechanism(s) = > Outcome] will be designed with Microsoft Visio 2016®. The final report will outline the quality of the empirical data used to test the final NPIS feedback theory and the limitations of such tests.

### Expert consultations

Discussions will be planned between the research team and two expert panels to verify whether the NPIS feedback theory developed in this research makes sense in light of their experience [36]. The first group will include experts from our professional network and we will approach the Advisory Council on Quality Care and Patient Safety of the Secrétariat international des infirmières et infirmiers de l’espace francophone (SIDIIEF) for additional references to relevant experts. The second group will include stakeholders from a Swiss teaching hospital where an NPIS has been in place for 1 year. This expert consultation group will include a nurse researcher, a nurse project manager, a senior nurse, a clinical nurse specialist, a nursing assistant and a physiotherapist or physician.

#### Step 6: Dissemination plans

We will share the results of this rapid realist review with experts and stakeholders. We will also publish this research in a peer-reviewed journal and will present the results at an international conference. Lastly, we will use this groundwork to conduct a realist evaluation of the feedback interventions currently deployed in the above-mentioned Swiss teaching hospital NPIS.

## Discussion

To our knowledge, our proposed combination of ANT with a realist methodology is original. These approaches hold promise for the identification of mechanisms in complex cyber-social systems and their various interactions (CM). They also offer new insights into the development of explanatory equations on feedback system which, to date, fail to provide convincing explanations.

Specifically, a realist approach to NPIS claims that interactions between mechanisms and contextual dynamics can explain outcomes (whether observed or not, planned or unexpected) of feedback system [36, 40]. It is therefore essential to explore and describe the various structures and actors involved, as well as their interactions and environment, in this complex cyber-social system [36]. In particular, interactions between entities (social or technical) could trigger hidden mechanisms induced by the conjunction of their internal structural properties [52]. The activation of mechanisms, however, also requires a particular context to generate outcomes or changes [36], amongst its own network of structures and mechanisms. This being said, complete explanation of a complex cyber-social system, either as programme or intervention, is not possible. Mechanisms must be prioritised according to their capacity (e.g. pertinence and consistency) to ensure sufficient explanatory power in the search for an operational theory [36]. In addition, causal chains should allow for a logical sequence of mechanisms [53].

Otherwise, given that our quest is to explain how feedback shared with interprofessional teams induces transformations and adaptations, ANT is particularly useful to inquire into NPIS entities, changes in (inter)actions and network reconfigurations. NPIS cyber-social systems can be examined as made up of intermediaries, actors, networks, translations and mediators [54].

Specifically, Callon [55] identifies four types of intermediaries that we can identify in NPIS: literary entries (texts, norms), technical artefacts (dashboards, electronic health records), human beings (skills, knowledge, and expertise), and resources (value and exchange instruments). Usually hybrid, intermediaries combine several types, and convey meaning to the system [55]. Actors refer to entities that bring together intermediaries. They can be organisations, groups of humans or assemblages of non-humans and “ (…) are defined through interactions – in the intermediaries they release” ([55], p., 135). Networks are thus comprised of intermediaries or actors that define each other in their intra- and extra-network interactions [55]. NPIS contain both intermediaries and actors that interact as they engage in action. According to Callon [55], actions are inherent to the creation or release of intermediaries which, in our view, could reflect the emergence of mechanisms and system changes.

Conceptualisations of such mechanisms can be further refined. For recall, we have referred to translation processes in our description of our data extraction form (Step 4, above). “Translation is the process by which networks are created, expand, and act” ([23], p., 176). It incorporates four non-linear phases, that we can interpret as possible mechanisms in NPIS: problematisation (actors identify problems or issues [upon feedback]), interessement (actors elaborate strategies and engage others in resolving problems/issues), enrolment (actors define and interrelate their roles to match their interests) and mobilisation (a critical mass of actors becomes capable of coordinating their efforts to act together) [23]. The actors that can move other people, to proceed with translation, are called mediators. These strategic actors modify and create connections that reshape and change networks, through negotiations and actions at the heart of translation processes [23]. We suggest that mediators engage in critical passageways that structure network (system) transformations or adoption of innovative interventions. We postulate that two key concepts related to these critical passageways, controversy and convergence Callon [55], are crucial to the study of mechanisms in NPIS feedback interventions.

For reference, “Controversies [tie] together and enmesh the techno-scientific and political contents that make up the issues facing actors” ([23], p., 176). Without doubt, feedback interventions uncover stimulating controversies [17]. Convergence is defined as

“(…) the closure of controversies among actors that creates agreement among them and strengthens the network, stabilising the [system]. Controversies are solved through translation by the addition of knowledge, other viewpoints and argumentative elements, as well as by the strengthening of existing connections and the enrolment of relevant new actors bringing new knowledge and resources necessary for action” ([23], pp., 176–177).

The above-mentioned ANT concepts are elegant cognitive devices to engage in realist methodology abductive thinking processes, particularly to identify mechanisms. In addition, context can also be described with reference to the same ANT concepts: intermediaries, actors, mediators, or network can influence the mechanisms at play. However, further refinements should be considered when inquiring into a specific step of NPIS, since feedback can be contextualised within a wider sequence of processes [27, 56]. Minary et al. [57] ingeneously suggest that context can be defined as endogenous and exogenous. The first is constituted of dense and stable inter-related entities within a system, programme or intervention [57]. Otherwise, the exogenous context is characterised by less stable and dense connections between entities [57]. Sure enough, exogenous entities can eventually be connected to endogenous entities through the action of mediators [57]. This underscores the crucial influence of chronology, particularly in the case of feedback processes that can evolve over time and become more effective in NPIS [25]. In this way, both ANT methodology, as suggested by Bilodeau and Potvin [23], and the transitive domain of the realist theory [21] concur.

In short, this realist review will propose an operational theory that will fill an enduring knowledge gap in NPIS. It will provide a framework to explain how feedback works, in which context, and what its outcomes are. Although rapid in terms of temporal duration, this review is an essential step prior to embarking on a realist evaluation of an ongoing innovative NPIS. We offer this review protocol as an exercise to demonstrate that the combination of an appropriate theory, such as ANT, with a realist methodology provides guidance and orientation to “rapidly” proceed, with rigor and creativity.

## Additional file

Additional file 1: The PRISMA-P 2015 checklist. (DOCX 30 kb)

## Data Availability

The data used and/or analysed during the current study are available from the corresponding author upon reasonable request.

## Abbreviations

ANT: Actor-Network Theory
NPIS: Nursing performance improvement systems
